# Human heme oxygenase 1 is a potential host cell factor against dengue virus replication

**DOI:** 10.1038/srep32176

**Published:** 2016-08-24

**Authors:** Chin-Kai Tseng, Chun-Kuang Lin, Yu-Hsuan Wu, Yen-Hsu Chen, Wei-Chun Chen, Kung-Chia Young, Jin-Ching Lee

**Affiliations:** 1 Institute of Basic Medical Sciences, College of Medicine, National Cheng Kung University, Tainan, Taiwan; 2 Center of Infectious Disease and Signaling Research, College of Medicine, National Cheng Kung University, Tainan, Taiwan; 3 Doctoral Degree Program in Marine Biotechnology, College of Marine Sciences, National Sun Yat-Sen University, Kaohsiung, Taiwan; 4 Division of Infectious Diseases, Department of Internal Medicine, Kaohsiung Medical University Hospital, Kaohsiung, Taiwan; 5 School of Medicine, Graduate Institute of Medicine, Sepsis Research Center, Center for Dengue Fever Control and Research, Kaohsiung Medical University, Kaohsiung, Taiwan; 6 Department of Biological Science and Technology, College of Biological Science and Technology, National Chiao Tung University, HsinChu, Taiwan; 7 Center for Dengue Fever Control and Research, Kaohsiung Medical University, Kaohsiung, Taiwan; 8 Graduate Institute of Medicine, College of Medicine, Kaohsiung Medical University, Kaohsiung, Taiwan; 9 Department of Medical Laboratory Science and Biotechnology, College of Medicine, National Cheng Kung University, Tainan, Taiwan; 10 Department of Biotechnology, College of Life Science, Kaohsiung Medical University, Kaohsiung, Taiwan; 11 Graduate Institute of Natural Products, College of Pharmacy, Kaohsiung Medical University, Kaohsiung, Taiwan; 12 Research Center for Natural Products and Drug Development, Kaohsiung Medical University, Kaohsiung, Taiwan

## Abstract

Dengue virus (DENV) infection and replication induces oxidative stress, which further contributes to the progression and pathogenesis of the DENV infection. Modulation of host antioxidant molecules may be a useful strategy for interfering with DENV replication. In this study, we showed that induction or exogenous overexpression of heme oxygenase-1 (HO-1), an antioxidant enzyme, effectively inhibited DENV replication in DENV-infected Huh-7 cells. This antiviral effect of HO-1 was attenuated by its inhibitor tin protoporphyrin (SnPP), suggesting that HO-1 was an important cellular factor against DENV replication. Biliverdin but not carbon monoxide and ferrous ions, which are products of the HO-1 on heme, mediated the HO-1-induced anti-DENV effect by non-competitively inhibiting DENV protease, with an inhibition constant (Ki) of 8.55 ± 0.38 μM. Moreover, HO-1 induction or its exogenous overexpression, rescued DENV-suppressed antiviral interferon response. Moreover, we showed that HO-1 induction by cobalt protoporphyrin (CoPP) and andrographolide, a natural product, as evidenced by a significant delay in the onset of disease and mortality, and virus load in the infected mice’s brains. These findings clearly revealed that a drug or therapy that induced the HO-1 signal pathway was a promising strategy for treating DENV infection.

The global burden of dengue virus (DENV) infection is high because of its transmission through efficient mosquito vectors across many tropical and subtropical countries worldwide. Approximately 400 million people are infected with DENV every year across approximately 100 countries, with a potential for further transmission^1^. Symptoms of DENV infection range from acute self-limiting febrile illness to life-threatening dengue hemorrhagic fever (DHF) and dengue shock syndrome (DSS)^2^. An effective vaccine or therapeutic agent for treating DENV infection is unavailable to date. Therefore, there is a high need to identify a potential viral or host target for developing an anti-DENV drug.

DENV is an enveloped, positive-stranded RNA virus belonging to the family *Flaviviridae*^3^. Antigenic diversity of DENV because of the absence of long-term cross-immunity among its 4 serotypes (DENV 1–4) allows it to induce multiple sequential infections^4^,^5^. DENV has an 11-kb genome that encodes a single polyprotein that is cleaved by both host and viral NS2B/NS3 protease into at least 10 mature proteins: 3 structural proteins (C, prM, and E) and 7 nonstructural proteins (NS1, NS2A, NS2B, NS3, NS4A, NS4B, and NS5)^6^. In addition, the DENV NS2B/NS3 protease inhibits the host type I interferon (IFN) pathway by cleaving a human mediator of IRF3 activation (MITA) to promote the progression of DENV infection^7^.

Type I IFNs, including IFN-α and IFN-β, play a critical role in protecting the human body against viral infections^8^,^9^. Both *in vitro* and *in vivo* studies indicate that DENV replication is sensitive to IFNs^10^,^11^. Furthermore, downstream signaling molecules involved in the IFN pathway, including, 2′-5′-oligoadenylate synthetase/RNase L and PKR, contribute to host defense against a DENV infection^12^,^13^,^14^. Therefore, the DENV has evolved a mechanism to inhibit the IFN signaling to aid its survival and replication in host cells^15^. DENV NS2B/NS3 protease plays an important role evading host innate immunity^7^. As mentioned previously, the DENV NS2B/NS3 protease inhibits the host type I IFN pathway by cleaving human mediator of IRF3 activation (MITA), thus preventing it from activating RIG-I and IRF3^7^.

DENV infection and replication increases the oxidative stress leading to activation of several inflammatory regulators, such as NFκB and C/EBPβ that caused severe damage of DENV-infected cells^16^,^17^,^18^. More recently, some reports indicated that microRNAs (miRNAs) were also play an important role in compromising DENV-induced oxidative stress and maintaining redox homeostasis^19^. Clinically, the collapsing redox homeostasis and damage induced by oxidative stress have been correlated with sever dengue-associated disease patients, suggesting that oxidative stress may responsible for DENV-induced pathogenesis^20^,^21^,^22^. The generated reactive oxygen species (ROS) is eliminated by antioxidant molecules such as heme oxygenase-1 (HO-1). HO-1 is an inducible enzyme in heme catabolic pathway that exerts protective effects against oxidative stress^23^. HO-1 degrades heme to produce biliverdin, carbon monoxide (CO) and ferrous iron (Fe^3+^) that function as potential cytoprotectants^24^,^25^. Recent evidences indicate that upregulation of HO-1 expression or overproduction of its metabolites suppresses hepatitis C virus (HCV) replication by activating Nrf2/Keap1 or by suppressing Bach1^23^,^26^. Biliverdin inhibits viral replication by increasing IFN response and by inhibiting HCV NS3/4A protease activity^27^,^28^. In addition, our recent studies indicate that small molecular compounds having HO-1 induction activity can be used as potential anti-HCV agents^23^,^26^. HO-1 induction or overexpression also exerts similar antiviral effects on HIV and HBV infections^29^,^30^.

We showed that HO-1 induction significantly inhibited DENV replication and that HO-1 attenuation by using its specific inhibitor tin protoporphyrin (SnPP) promoted DENV replication, suggesting that HO-1 was an important cellular factor for affecting DENV replication. This anti-DENV effect of HO-1 was mediated by biliverdin that restored DENV-suppressed host antiviral IFN response by non-competitively inhibiting DENV NS2B/NS3 protease. HO-1 induction in DENV-infected ICR suckling mouse model delays DENV-2-induced lethality. Furthermore, a nature compound andrographolide inhibited DENV both *in vitro* and *in vivo* by inducing HO-1 expression. These findings can be used to design anti-DENV drugs or therapies.

## Result

### HO-1 induction inhibits DENV protein synthesis and RNA replication

**Figure 1:**
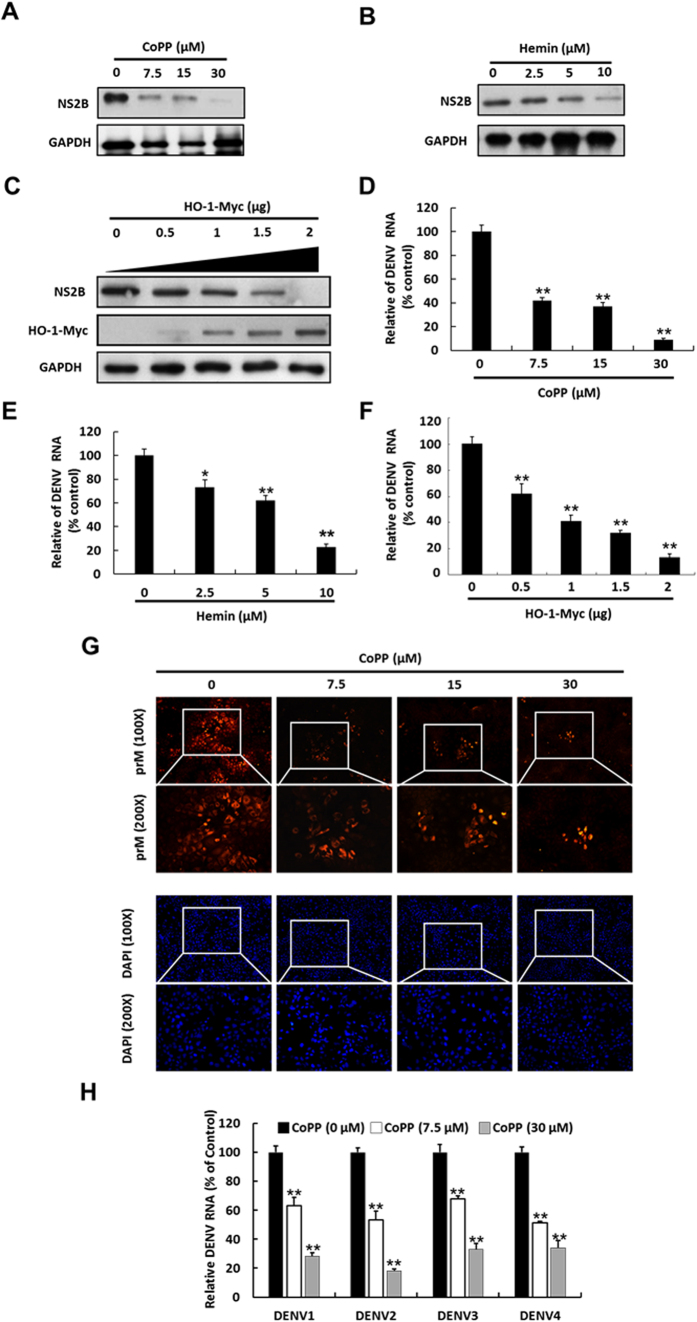
HO-1 inhibits DENV protein synthesis and RNA replication. Inhibition of DENV protein synthesis by HO-1 inducer (**A**) CoPP, (**B**) hemin or (**C**) transient expression of exogenous HO-1 in a dose-dependent manner. DENV-infected Huh-7 cells were treated with CoPP and hemin or were transfected with a vector that transiently expressed exogenous HO-1 at indicated concentrations for 3 days. Western blotting was performed to determine DENV protein synthesis in the infected Huh-7 cells treated with CoPP or hemin or transiently expressing exogenous HO-1. GAPDH was used as an equal loading control. Inhibition of DENV RNA replication by HO-1 inducer (**D**) CoPP or (**E**) hemin or (**F**) by the transient expression of exogenous HO-1 in a dose-dependent manner. DENV RNA level was analyzed by performing RT-qPCR, were normalized against the mRNA level of cellular *gapdh*, and was presented as percentage change relative to that in DENV-infected Huh-7 cells (defined as 100%). (**G**) Representative immunofluorescence images showing CoPP-induced inhibition of DENV replication in DENV-infected Huh-7 cells. DENV-infected Huh-7 cells were treated 0–30 μM CoPP for 3 days, fixed, and examined by performing the immunofluorescence assay with anti-DENV prM antibody, and stained with DAPI. (**H**) CoPP inhibited the replication of DENV serotypes 1–4. Huh-7 cells were infected with the 4 DENV serotypes and were treated with CoPP at indicated concentration for 3 days. RNA level of DENV serotypes 1–4 were analyzed by performing RT-qPCR, were normalized against the mRNA level of cellular *gapdh*, and were presented as percentage change relative to that in DENV-infected Huh-7 cells (defined as 100%). Results are expressed as the mean ± SD (error bar) of 3 independent experiments; *P < 0.05, **P < 0.01.

**Figure 2:**
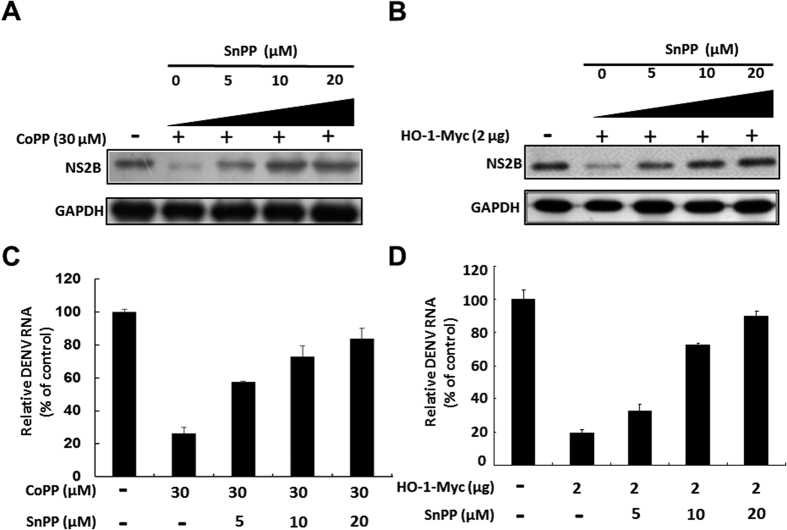
HO-1-specific inhibitor SnPP attenuates the inhibitory effect of HO-1 induction on DENV replication. SnPP attenuates the inhibitory effect of (**A**) HO-1 inducer and (**B**) transient expression of exogenous HO-1 on DENV protein synthesis. DENV-infected Huh-7 cells were treated with 30 μM CoPP or with 2 μg vector that transiently expressed exogenous HO-1, followed by treatment with 0–20 μM SnPP for 3 days. Total cell lysate was collected for performing western blotting to analyze DENV protein synthesis. Levels of GAPDH were used as equal loading control. SnPP attenuates the inhibitory effect of (**C**) HO-1 inducer and (**D**) transient expression of exogenous HO-1 on DENV RNA replication. Huh-7 cells were treated with 30 μM CoPP or with 2 μg of the vector that transiently expressed exogenous HO-1, followed by treatment with 0–20 μM SnPP for 3 days. Total cellular RNA was collected for performing RT-qPCR to analyze DENV RNA replication. The mRNA level of *gapdh* was used as the equal loading control. Results are expressed as the mean ± SD (error bar) of 3 independent experiments; *P < 0.05, **P < 0.01.

In the present study, we have observed that DENV infection induces oxidative stress, leading to a gradual activation of antioxidant molecule HO-1 in promoter activity and protein synthesis at early time point, from 6 to 12 hrs. However, HO-1 promoter activity and protein synthesis were markedly decreased by DENV infection at later time point, from 24 to 72 hrs. (Figure S1). Based on this observation, we hypothesized that the elevated HO-1 expression or activity should be harmful for DENV replication. To examine whether HO-1 induction exhibited anti-DENV activity, we treated DENV-infected Huh-7 cells with HO-1-specific inducers CoPP and hemin at indicated concentrations or transiently overexpressed HO-1 for 3 days. DENV protein synthesis and RNA replication were analyzed by performing western blotting and RT-qPCR, respectively. In addition, the cytotoxic effects were tested by performing MTS assay (Figure S2). Our results showed that CoPP and hemin treatment and HO-1 overexpression dramatically decreased DENV protein synthesis in a dose-dependent manner (Fig. 1A–C). RT-qPCR also showed the inhibitory effect of CoPP and hemin treatment and HO-1 overexpression on DENV RNA replication (Fig. 1D–F). We also performed immunofluorescence assay to confirm the anti-DENV effects of HO-1 induction. Our results showed that CoPP decreased DENV PrM protein expression in DENV-infected Huh-7 cells in a dose-dependent manner compared with that in CoPP-untreated cells (Fig. 1G). Furthermore, HO-1 induction inhibited DENV serotypes 1–4 (Fig. 1H). To further confirm the anti-DENV effects of HO-1 induction, we used an HO-1-specific inhibitor SnPP to attenuate HO-1 induction by CoPP treatment or its transient overexpression. SnPP dose-dependently attenuated the anti-DENV effects exerted by CoPP treatment and by the transient overexpression of HO-1 on DENV protein synthesis compared with that on SnPP-untreated controls (Fig. 2A,B). RT-qPCR showed similar results for DENV RNA replication under the same experimental condition as described above (Fig. 2C,D).

### Biliverdin inhibits DENV replication but CO and Fe^3+^ do not

**Figure 3:**
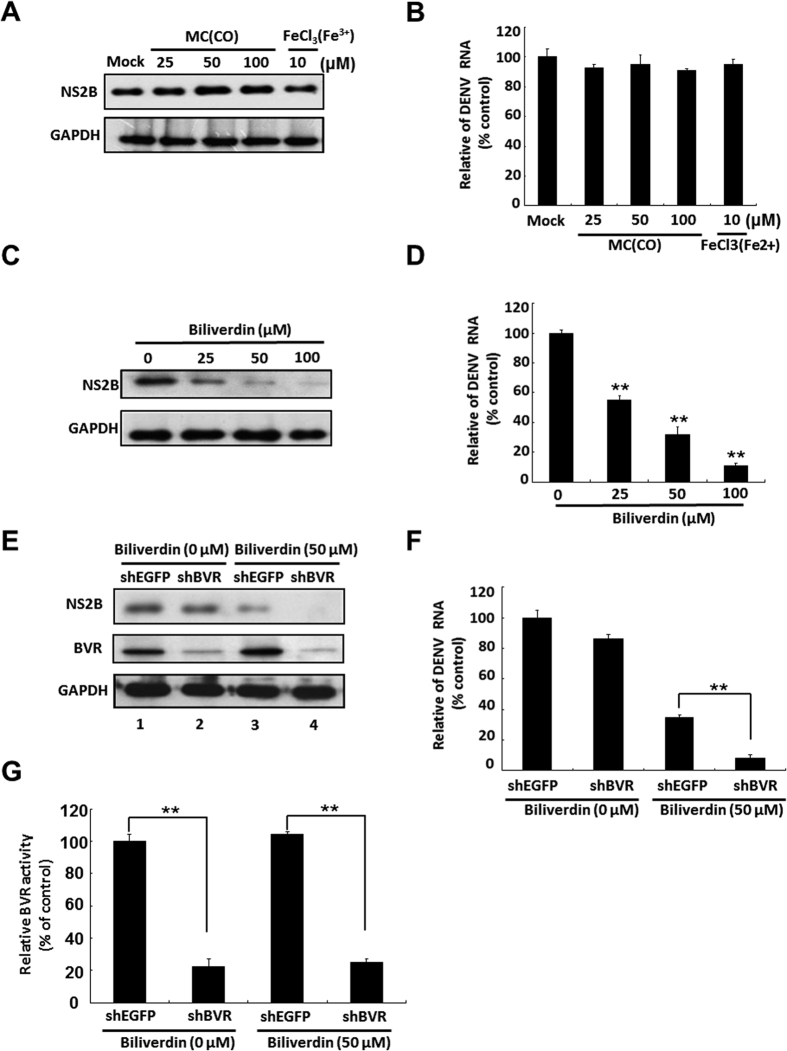
Biliverdin but not CO and Fe^3+^ inhibits DENV replication. Effect of (**A**) CO, Fe^3+^, and (**B**) biliverdin on DENV protein synthesis. Western blotting was performed to determine DENV protein synthesis in DENV-infected Huh-7 cells treated with CO donor MC, Fe^3+^ donor FeCl_3_, or biliverdin at indicated concentrations for 3 days. GAPDH was used as the equal loading control. Effect of (C) CO, Fe^3+^, and (**D**) biliverdin on DENV RNA replication. DENV-infected Huh-7 cells were treated with CO donor MC, Fe^3+^ donor FeCl_3_, or biliverdin at indicated concentrations for 3 days. DENV RNA level was analyzed by performing RT-qPCR, were normalized using the mRNA level of cellular *gapdh*, and was presented as percentage change relative to that in DENV-infected Huh-7 cells (defined as 100%). Effects of silencing the gene encoding BVR on DENV (**E**) protein synthesis (**F**) RNA replication. (**G**) BVR activity. Huh-7 cells were transfected with shBVR or shEGFP (control). After 6 h, the transfected cells were infected with 0.2 MOI DENV-2 strain 16681 for 2 h at 37 °C and were incubated with medium with or without 50 μM biliverdin for 3 days. Total cell lysate was analyzed by performing western blotting with anti-NS2B, anti-BVR, and anti-GAPDH (loading control) antibodies. Total cellular RNA was extracted for performing RT-qPCR with specific primers against DENV NS5 gene and *gapdh* (loading control). BVR activity was measured using a BVR assay kit and was calculated from a standard curve generated using a biliverdin-positive control solution. BVR activity is presented as percentage relative to that in shEGFP-treated and biliverdin-untreated cells (defined as 100%). Results are expressed as the mean ± SD (error bar) of 3 independent experiments; *P < 0.05, **P < 0.01.

HO-1 degrades heme to biliverdin, CO, and Fe^3+^ 31. To determine whether these products mediated the anti-DENV activity of HO-1, we incubated DENV-infected cells with indicated concentrations of CO donor methylene chloride (MC), Fe^3+^ donor FeCl_3_, or biliverdin for 3 days. In addition, cytotoxic effects of these compounds were determined by performing MTS assay. Treatment of DENV-infected cells with MC and iron III chloride did not interfere with DENV protein synthesis and RNA replication (Fig. 3A,B). However, treatment of these cells with biliverdin decreased DENV protein synthesis and RNA replication in a dose-dependent manner (Fig. 3C,D), without inducing cytotoxic effects (Figure S3). These results indicated that biliverdin but not by CO and Fe^3+^ mediated the inhibitory effects of HO-1 induction on DENV replication. In the human body, biliverdin is rapidly reduced to bilirubin by biliverdin reductase (BVR)^32^. To determine whether bilirubin also exerted anti-DENV effects, we transfected cells with an shRNA against the gene encoding BVR (shBVR) to prevent the reduction of biliverdin to bilirubin, which resulted in the accumulation of biliverdin in these cells. Cells transfected with shBVR or EGFP-specific shRNA (shEGFP) were infected with DENV and were incubated in a culture medium with or without 50 μM biliverdin. The cells were harvested, and DENV protein synthesis and RNA replication were determined by performing western blotting and RT-qPCR, respectively. DENV protein levels were not significantly different between shBVR- and shEGFP-transfected cells not treated with biliverdin (Fig. 3E, top panel, lanes 1 and 2). However, shBVR-transfected cells treated with biliverdin showed enhanced anti-DENV activity compared with shEGFP-transfected cells treated with biliverdin (Fig. 3E, top panel, lanes 3 and 4). Similar results were obtained for DENV RNA replication under the same experimental conditions described above (Fig. 3F). To confirm the efficiency of shBVR under the same experimental conditions as those described above, we examined the level and activity of BVR in shBVR-transfected and shBVR-untransfected cells. The protein level and activity of BVR were significantly decreased in shBVR-transfected cells (Fig. 3E, middle panel, and G). These results indicated that biliverdin and not bilirubin contributed to the anti-DENV effect of HO-1 induction.

### Biliverdin inhibits DENV NS2B/NS3 protease activity

**Figure 4:**
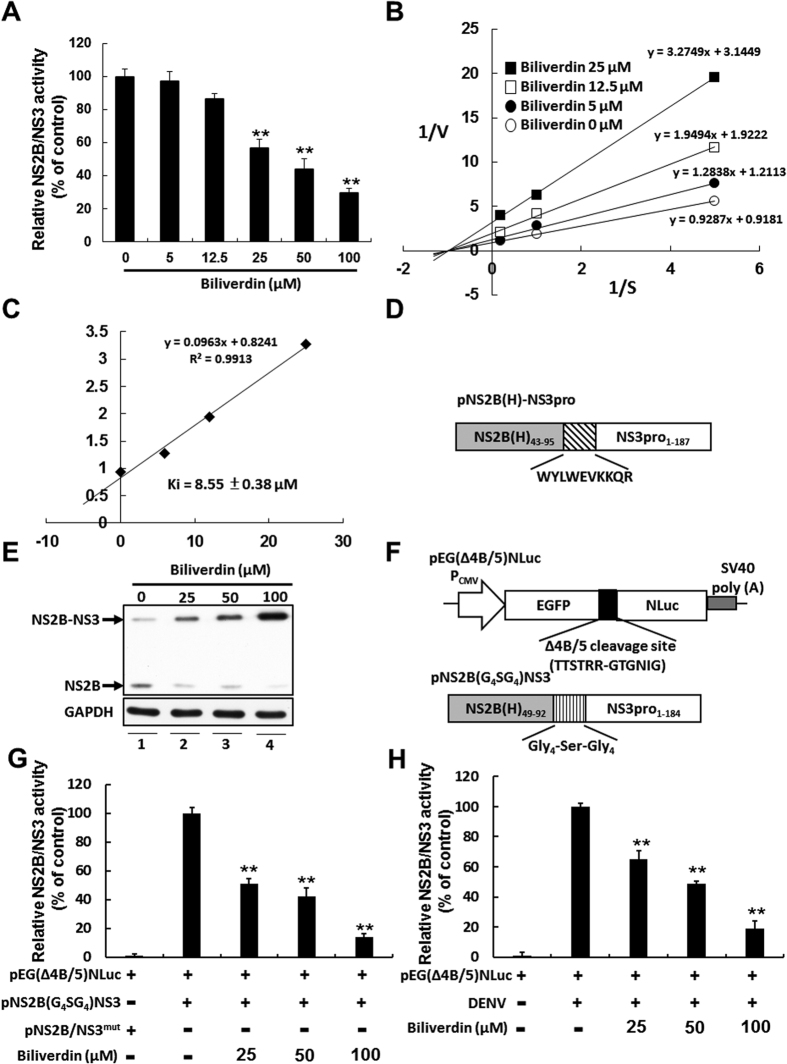
Biliverdin inhibits DENV NS2B/NS3 protease activity. (**A**) Inhibition of DENV protease activity by biliverdin. DENV protease activity was determined by incubating the indicated concentrations of biliverdin with recombinant DENV protease and Boc-GRR-AMC in a cleavage buffer for 30 min. Free AMC was measured using a spectrofluorometer and was expressed as percentage change relative to that in biliverdin-untreated controls (defined as 100%). (**B,C**) Determination of mechanisms underlying the effect of biliverdin on DENV NS2B/NS3 protease activity. The fluorogenic peptide at indicated concentrations was incubated with recombinant DENV protease in the presence of 5, 12.5 and 25 μM biliverdin for 30 min. The rate of release of AMC as a function of DENV protease activity was determined using a spectrofluorometer. Kinetic parameters and mode of inhibition were calculated from a standard curve generated using an AMC-positive control solution and were analyzed using Lineweaver–Burk plots of reciprocal velocity at the indicated concentrations of biliverdin. (**D**) Schematic diagram of the NS2B/NS3 protease vector with autocleavage activity (**E**) Inhibition of cis-acting activity of DENV protease by biliverdin in Huh-7 cells. The pNS2B(H)-NS3pro-transfected Huh-7 cells were treated with biliverdin at increasing concentrations for 3 days. The western blotting analysis were performed using anti-DENV NS2B specific antibody. (**F**) Schematic diagram of the protease reporter vector and the protease vector (**G,H**) Inhibition of trans-acting activity of DENV protease by biliverdin in Huh-7 and DENV-infected Huh-7 cells. The protease reporter vector and protease expression vector were co-transfected into Huh-7 cells or protease reporter vector alone was transfected into DENV-infected Huh-7 cells, followed by incubation of biliverdin at increasing concentrations for 3 days. Each transfection mixture contained 0.1 μg of firefly luciferase expression vector as a transfection control for normalization against the nano luciferase activity. Transfection of dysfunctional NS2B/NS3 protease expression vector or non-DENV-infected Huh-7 cells served as a negative control. The trans-acting activity of DENV protease is presented as percentage change relative to that in wild type protease-transfected or DENV-infected Huh-7 cells and biliverdin-untreated cells (defined as 100%). Results are expressed as the mean ± SD (error bar) of 3 independent experiments; *P < 0.05, **P < 0.01.

Bile pigments inhibit serine-activated protease such as chymotrypsin, trypsin, and HCV NS3 protease^28^. To investigate whether biliverdin inhibited DENV NS2B/NS3 protease activity, we measured the effect of biliverdin on DENV protease by performing an enzyme-based fluorescence peptide cleavage assay. This assay was conducted using fluorescent peptide Boc-GRR-AMC, recombinant DENV NS2B/NS3 protease, and increased concentrations of biliverdin. As shown in Fig. 4A, biliverdin inhibited DNEV NS2B/NS3 protease in a concentration-dependent manner. Next, we determined the kinetics of biliverdin-induced inhibition of DENV NS2B/NS3 protease by using Lineweaver-Burk plots. On the basis of the plots’ characteristics, different biliverdin concentration plots were intersection at the X-axis, which indicated that biliverdin non-competitively inhibited DENV NS2B/NS3 protease (Fig. 4B). Each biliverdin concentration versus the slope (Km/V) of primary reciprocal plots was used to prepare secondary plots to determine Ki. The plots showed highly significant linearity (r = 0.9913, p < 0.005; Fig. 4C) and indicated that biliverdin non-competitively inhibited DENV NS2B.NS3 protease, with a Ki of 8.55 ± 0.38 μM.

To confirm the inhibitory effect of biliverdin on NS2B/NS3 protease activity in the intracellular condition, we first examine the cis-acting activity of DENV protease in Huh-7 cells. The Huh-7 cells were transfected with 1 μg of DENV NS2B/NS3 protease vector pNS2B(H)-NS3pro with autocleavage activity (Fig. 4D), the transfected-cells were treated with biliverdin at increasing concentrations for 3 days. The amount of uncleaved products upon biliverdin treatment were examined by western blotting analysis with anti-DENV NS2B specific antibody. As shown in Fig. 4E, biliverdin resulted in dose-dependent accumulation of uncleaved NS2B/NS3 protein level (lanes 2-3) compared with biliverdin-untreated sample (lane 1), indicating that biliverdin can block cis-acting activity of DENV protease. Furthermore, the trans-acting activity of DENV protease in Huh-7 cells were measured. The NS2B/NS3 protease reporter vector pEG(∆4B/5)NLuc and NS2B/NS3 protease expression vector pNS2B(G4SG4)NS3 without autocleavage activity (Fig. 4F) were co-transfected into Huh-7 cells, followed by incubation of biliverdin at increasing concentrations for 3 days. As shown in Fig. 4G, biliverdin dose-dependently decreased the trans-acting activity of DENV protease through luciferase activity assay. Similarly, biliverdin dose-dependently decreased the trans-acting activity of DENV protease in pEG(∆4B/5)NLuc-transfected Huh-7 cells under DENV infection through luciferase activity assay (Fig. 4H).

### HO-1 induction rescues DENV protease-suppressed antiviral IFN response

**Figure 5:**
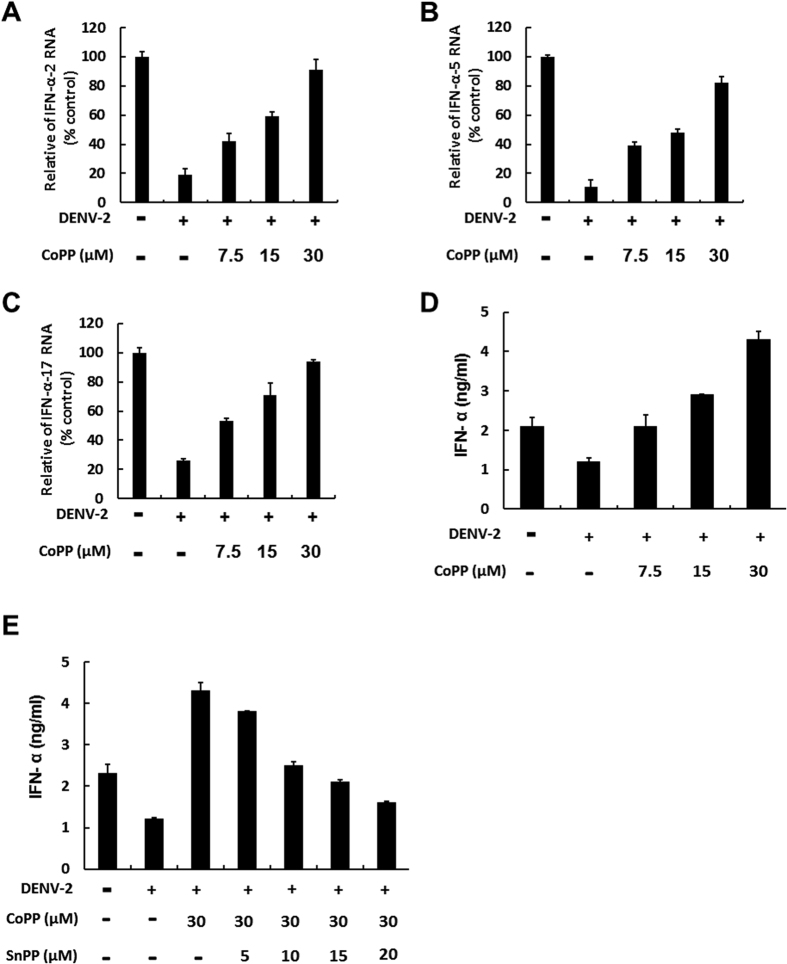
HO-1-specific inducer CoPP rescues DENV protease-suppressed IFNα production in DENV-infected Huh-7 cells. CoPP rescues DENV protease-suppressed transcription of genes encoding (**A**) IFN-α-2, (**B**) IFN-α-5, and (**C**) IFN-α-17 in DENV-infected Huh-7 cells. DENV-infected Huh-7 cells were treated with 7.5, 15, and 30 μM CoPP for 3 days. Total cellular RNA was extracted for performing RT-qPCR with specific primers against the genes encoding IFN-α-2, IFN-α-5, and IFN-α-17. The mRNA level of these genes were normalized against that of cellular *gapdh* and were presented as percentage change relative to that in parental Huh-7 cells (defined as 100%). (**D**) CoPP rescues DENV protease-suppressed IFN-α protein expression in DENV-infected Huh-7 cells. DENV-infected Huh-7 cells were treated with 7.5, 15, and 30 μM CoPP for 3 days. The culture fluid was examined by performing ELISA to determine IFN-α protein levels. (**E**) SnPP attenuates the CoPP-induced expression of IFN-α in DENV-infected Huh-7 cells. DENV-infected Huh-7 cells were coincubated with 30 μM CoPP and increasing concentrations of SnPP for 3 days. The culture fluid was examined by performing ELISA to determine IFN-α protein levels. Results are expressed as the mean ± SD (error bar) of 3 independent experiments; *P < 0.05, **P < 0.01.

**Figure 6:**
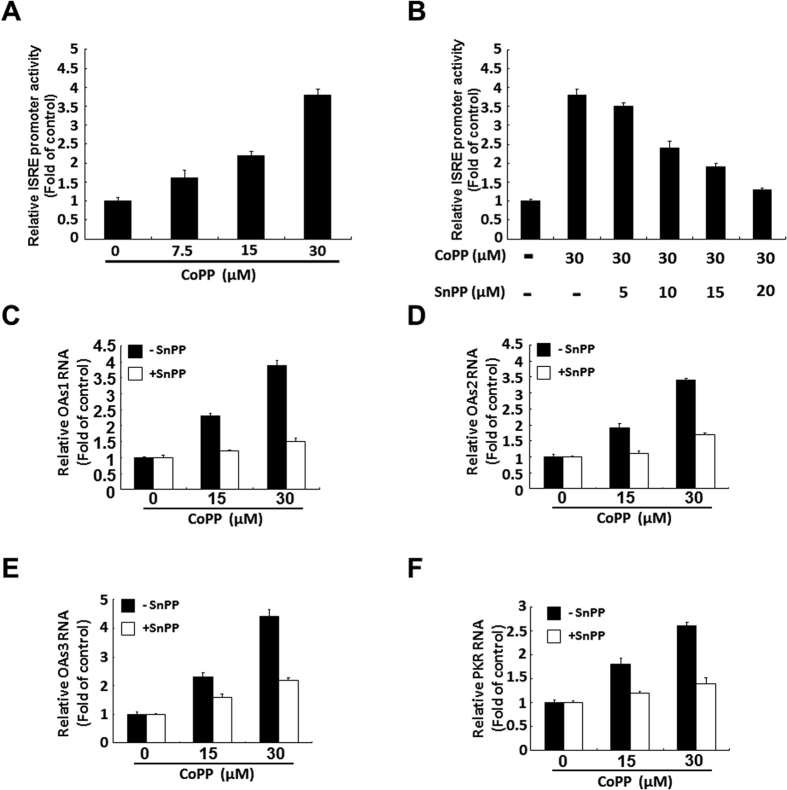
HO-1-specific inducer CoPP induces antiviral IFN responses in DENV-infected Huh-7 cells. (**A**) CoPP induces the ISRE promoter activity in DENV-infected Huh-7 cells. Huh-7 cells were transfected with pISRE-Luc. After 6 h, the transfected cells were infected with 0.2 MOI DENV-2 strain 16681 for 2 h at 37 °C and were treated with 7.5, 15, and 30 μM CoPP for 3 days. Luciferase activity was measured to determine the ISRE promoter activity and was presented as fold activation relative to that in CoPP-untreated cells (defined as 1). (**B**) SnPP attenuates CoPP-induced stimulation of the ISRE promoter activity in DENV-infected Huh-7 cells. Huh-7 cells were transfected with pISRE-Luc plasmid. After 6 h, the transfected cells were infected with 0.2 MOI DENV-2 strain 16681 for 2 h at 37 °C and were treated with 30 μM CoPP and increasing concentrations of SnPP for 3 days. Luciferase activity was measured to determine the ISRE promoter activity and was presented as fold activation relative to that in CoPP-/SnPP-untreated cells (defined as 1). CoPP increases mRNA level of (**C**) OAS1, (**D**) OAS2, (**E**) OAS3 and (**F**) PKR, which are attenuated by SnPP in DENV-infected Huh-7 cells. DENV-infected Huh-7 cells were treated with 15 and 30 μM CoPP in the presence or absence of 20 μM SnPP for 3 days. Total cellular RNA was extracted for performing RT-qPCR with specific primers against genes encoding OAS1-3 and PKR. The mRNA level of OAS1-3 and PKR were normalized against that of cellular *gapdh* and were presented as fold change relative to that in parental Huh-7 cells (defined as 1). Results are expressed as the mean ± SD (error bar) of 3 independent experiments.

Numerous studies have shown that DENV NS2B/NS3 protease inhibits the antiviral IFN response by cleaving MITA to disrupt the IFN synthesis pathway^7^,^33^. To determine whether the inhibitory effect of biliverdin on DENV protease rescued DENV protease-suppressed antiviral IFN response, which further enhanced its anti-DENV activity, we measured the expression of genes encoding IFN-α-2, IFN-α-5, and IFN-α-17 after treating DENV-infected cells with indicated concentrations of CoPP for 3 days. Parental Huh-7 cells showed basal expression of the abovementioned genes. As shown Fig. 5, CoPP rescued DENV-reduced mRNA expression of genes encoding IFN-α-2, IFN-α-5, and IFN-α-17 in a dose-dependent manner (Fig. 5A–C). As expected, we observed that CoPP also increased DENV-reduced IFN-α level in the supernatant in a dose-dependent manner (Fig. 5D), whereas SnPP treatment attenuated the CoPP-induced increase in DENV-reduced IFN-α level in a dose-dependent manner (Fig. 5E). To confirm whether HO-1 induction promoted downstream antiviral IFN response, we transfected DENV-infected cells with interferon-stimulated response element (ISRE)-driven firefly luciferase reporter plasmid and incubated these cells with CoPP for 3 days. CoPP significantly increased the ISRE promoter activity (Fig. 6A), as determined by measuring the luciferase activity. This inductive effect of CoPP on ISRE promoter activity was gradually eliminated after SnPP treatment (Fig. 6B). Next, we examined the expression of important IFN-stimulated genes (ISGs) such as those encoding 2′-5′-oligoadenylate synthetase 1 (OAS1), OAS2, OAS3, and PKR in response to DENV infection. Expression of ISGs was significantly increased after treatment with 15 and 30 μM CoPP, whereas SnPP treatment effectively attenuated inhibited CoPP-induced expression of ISGs (Fig. 6C–F). These data indicated that HO-1 induction rescued DENV protease-suppressed antiviral IFN response.

### HO-1 induction delays DENV-2-induced lethality in the suckling mouse model

**Figure 7:**
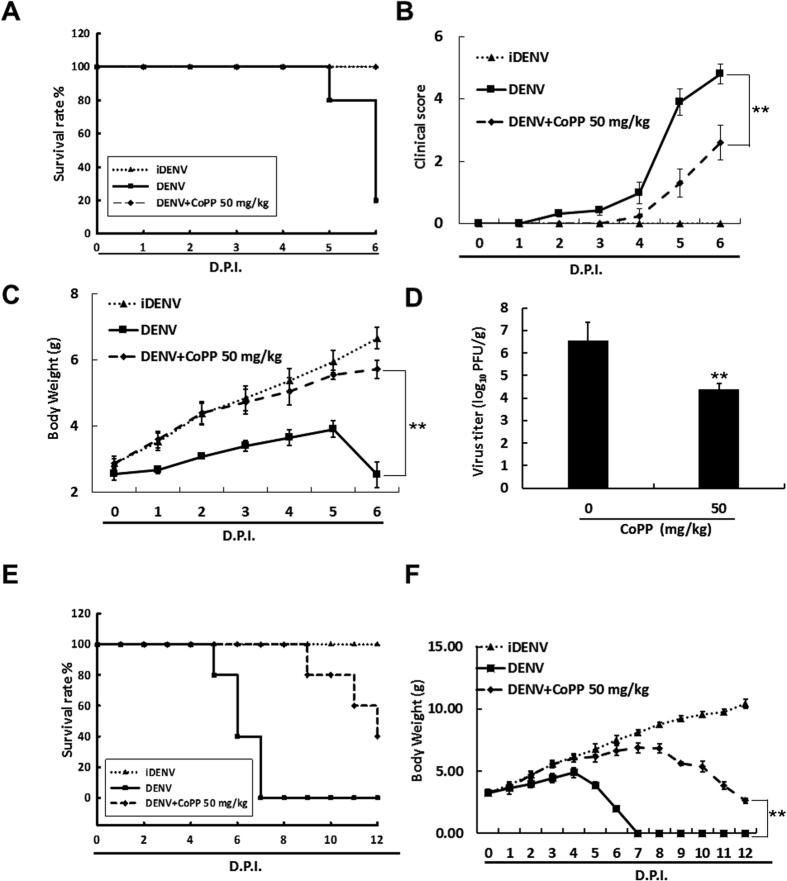
HO-1-specific inducer CoPP shows therapeutic efficacy in DENV-infected ICR suckling mice. Six-day-old ICR suckling mice were injected with 2.5 × 10^5^ pfu DENV-2 intracerebrally and with CoPP (50 mg/kg) intraperitoneally at 1, 3 and 5 dpi. (**A**) Survival rate, (**B**) clinical score, and (**C**) body weight of the mice were daily measured to 6 dpi. Illness symptoms were scored as follow: 0, no symptom; 1, slight weight loss and ruffled hair; 2, slow activity; 3, asthenia and anorexia; 4, paralysis and fatal illness; and 5, death. Next, 0.1 g of brain tissue of these mice was incubated in RPMI medium for viral titration. (**D**) DENV titer was determined by performing plague assay. DENV-infected ICR suckling mice were intraperitoneally injected with CoPP (50 mg/kg) at 1, 3, 5, 7 and 9 dpi. (**E**) Survival rate and (**F**) body weight of the mice were daily measured to 12 dpi. Mice in the control group were treated with 60 °C heat-inactive DENV for 30 min. Each group included 8–10 mice. Error bars indicate the mean ± SD of 3 independent experiments; *P < 0.05, **P < 0.01.

To investigate whether HO-1 induction exerted protective effects against DENV infection *in vivo*, 6-day-old ICR suckling mice were injected with DENV-2 intracerebrally and with HO-1 inducer CoPP (50 mg/kg) intraperitoneally at 1, 3, and 5 days post-infection (dpi). Mice injected with heat-inactive DENV-2 (iDENV) were used as mock controls. Survival rates and clinical scores of DENV-infected mice treated with or without CoPP were measured daily for 6 days. Subsequently, all the mice were sacrificed at 6 dpi, and their brain tissue were collected to measure viral titer and HO-1 induction. As shown in Fig. 7A, DENV-infected mice that were not treated with CoPP developed severe sickness leading to death within 4–6 dpi compared with iDENV-infected control mice. In contrast, CoPP at 50 mg/kg shielded 80% of the mice from the life-threatening effects of the DENV-2 infection when compared to the non-CoPP-treated mice. DENV-infected mice that did not receive CoPP developed severe paralysis, anorexia, and asthenia and lost 62.1% of their body weight compared with iDENV-treated mice at 6 dpi (Fig. 7B,C). In contrast, DENV-infected mice treated with CoPP showed slight paralysis, anorexia, and asthenia and lost only 14.2% of their body weight compared with iDENV-infected mice at 6 dpi (Fig. 7B,C). Furthermore, CoPP dramatically decreased the viral titer by 2.16 ± 0.4 log_10_ compared with that in DENV-infected mice not treated with CoPP (Fig. 7D). Furthermore, we observed that CoPP treatment extended life-span of DENV-infected mice more than 6 days when compared to DENV-infected mice without CoPP treatment following a longer period of time (Fig. 7E,F). We also observed that CoPP restored the DENV-reduced HO-1 protein synthesis (Figure S4A) and HO-1 mRNA level was increased by approximately 16 fold in DENV-infected mice treated with CoPP compared with that in DENV-infected mice not treated with CoPP (Figure S4B). Consistent with the cell-based results observed above, the mRNA level of IFN-α-2, IFN-α-5, OAS1, OAS2, OAS3, and PKR were increased in DENV-infected mice treated with CoPP compared with that in DENV-infected mice not treated with CoPP (Figure S5). These data indicated that HO-1 induction delays DENV-2-induced lethality and illness.

### Andrographolide inhibits DENV replication, induces HO-1 expression, and delays DENV-2-induced lethality in the suckling mouse model

**Figure 8:**
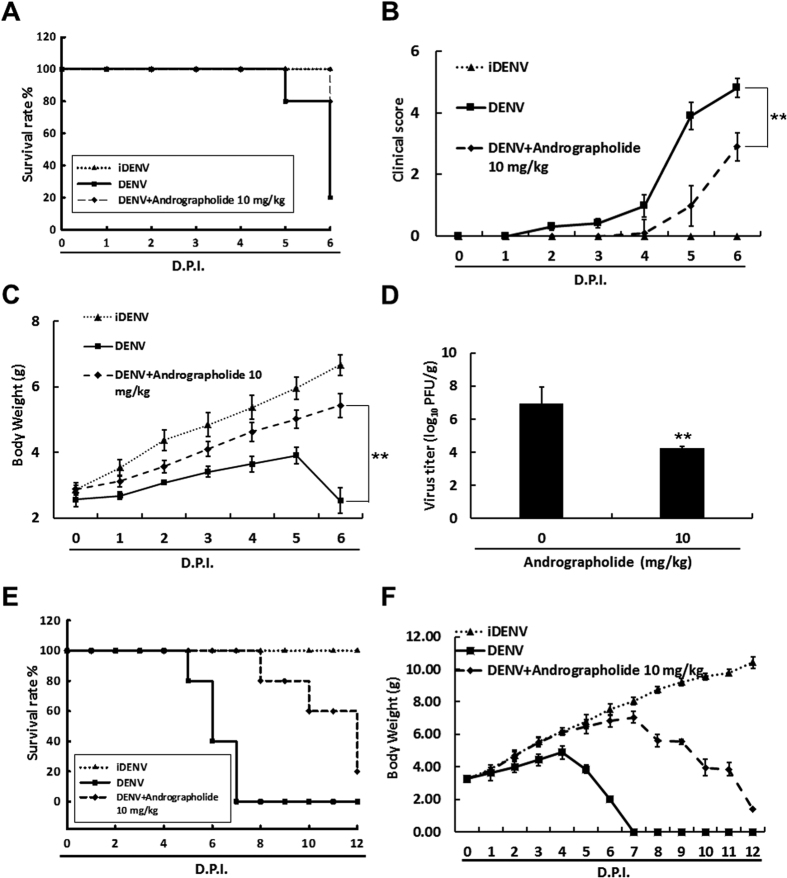
Andrographolide shows therapeutic efficacy in DENV-infected ICR suckling mice. Six-day-old ICR suckling mice were injected with 2.5 × 10^5^ pfu DENV-2 intracerebrally and with andrographolide (10 mg/kg) intraperitoneally at 1, 3, and 5 dpi. (**A**) Survival rate, (**B**) clinical score, and (**C**) body weight of the mice were daily measured to 6 dpi. Illness symptoms were scored as follows: 0, no symptoms; 1, slight weight loss and ruffled hair; 2, slowing of activity; 3, asthenia and anorexia; 4, paralysis and fatal illness; and 5, death. Next, 0.1 g brain tissue of these mice was incubated in an RPMI medium for viral titration. (**D**) A DENV titer was determined by performing the plague assay. DENV-infected ICR suckling mice were intraperitoneally injected with andrographolide (10 mg/kg) at 1, 3, 5, 7 and 9 dpi. (**E**) Survival rate and (**F**) body weight of the mice were daily measured to 12 dpi. Mice in the control group were treated with 60 °C heat-inactive DENV for 30 min. Each group included 8–10 mice.

Andrographolide is the most abundantly present diterpene lactone in the leaves and stems of *Andrographis paniculata* and is widely used herbal medicine for dietary supplement against many diseases^34^,^35^. In our previous study, we showed that andrographolide inhibited an HCV infection by upregulating HO-1 expression^23^. To confirm that HO-1 induction was a promising strategy for treating the DENV infection, we treated DENV-infected Huh-7 cells with andrographolide at indicated concentrations for 3 days. DENV protein synthesis was analyzed by performing western blotting. In addition, we determined the cytotoxic effect of andrographolide under the same experimental conditions by performing the MTS assay. Our results showed that andrographolide dramatically decreased DENV RNA replication and protein synthesis in a dose-dependent manner, without exerting any cytotoxic effect (Figure S6A–C). Moreover, HO-1 inhibitor SnPP attenuated the anti-DENV effect of andrographolide in a dose-dependent manner (Figure S6D). Next, we used the DENV-infected ICR suckling mouse model to investigate whether HO-1 induction exerted a protective effect against the DENV infection *in vivo.* Six-day-old ICR suckling mice were injected with DENV-2 intracerebrally and with andrographolide (10 mg/kg) intraperitoneally at 1, 3, and 5 dpi. Mice injected with iDENV were used as mock controls. As expected, treatment with 10 mg/kg andrographolide increased the survival rate of DENV-infected mice by 60% (Fig. 8A). At 6 dpi, andrographolide decreased illness and increased the body weight of DENV-infected mice by approximately 2.9 ± 0.54 g compared with those of DENV-infected mice not treated with andrographolide (Fig. 8B,C). In addition, andrographolide decreased the viral titer by 2.69 ± 0.4 log_10_ compared with that of DENV-infected mice not treated with andrographolide at 6 dpi (Fig. 8D). Similar to the observation of DENV-infected mice upon CoPP treatment described above, andrographolide treatment also extended life-span of DENV-infected mice more than 6 days when compared to DENV-infected mice without andrographolide treatment (Fig. 8E,F).

## Discussion

Our results indicated that HO-1 induction effectively suppressed the replication of the 4 DENV serotypes (Figs 1 and 2). HO-1 exhibits antiviral activity against HBV, HCV, and HIV even though mechanisms underlying the inhibition of these viruses are different^36^,^37^,^38^,^39^. To determine the precise mechanisms underlying the inhibition of DENV replication by HO-1 induction, we investigated the antiviral activities of products of HO-1 reaction. Our data indicated that biliverdin exerted potent antiviral effects against DENV infection (Fig. 3), as evidenced by the inhibition of DENV NS2B/NS3 protease in both enzyme-based and cell-based assays (Fig. 4). Moreover, antiviral activity of HO-1 induction in DENV-infected cells was accompanied with the reactivation of the antiviral IFN response (Figs 5 and 6). These results suggested that reactivation of the antiviral IFN response was associated with the inactivation of NS2B/NS3 protease by biliverdin, which prevented the cleavage of human MITA and innate immunity recognition sites, thereby restoring the signaling pathway for type I IFN synthesis. This observation is similar to the recent findings on HCV by Zhu *et al.*^28^. This possibility is being explored further in our ongoing investigations. Viral protease is an attractive target for antiviral therapies. Structurally, 180 residues at the N-terminus of DENV NS3 protease function similar to a trypsin or serine protease and require 40 residues of the hydrophilic domain of NS2B as cofactor^40^,^41^. Future studies should be performed to determine the functional group of biliverdin that contributes to its interaction with DENV protease to obtain more information for designing anti-DENV agents.

In the present study, we demonstrated that the HO-1 induction significant delays in the onset of disease and mortality, and virus load in the infected mice’s brains (Figs 7 and 8). HO-1 induction activated the antiviral IFN response in the DENV-infected ICR suckling mouse model (Figure S5). Chia-Yi Yu *et al.* have shown that DENV NS2B/NS3 protease recognizes and cleaves human MITA, thus disrupting the antiviral IFN response^7^. Our cell-based assay suggested that reactivation of the antiviral IFN response was associated with the inactivation of NS2B/NS3 protease by biliverdin. However, DENV NS2B/NS3 protease cannot recognize and cleave mouse mediator of IRF3 activation (STING)^7^. In addition, Lehmann *et al.* showed that biliverdin exhibited anti-HCV activity by increasing the antiviral IFN response^27^. Therefore, HO-1 induction may activate the antiviral IFN response as an alternative anti-DENV mechanism. The precise mechanisms should be investigated at the molecular level.

In recent years, many studies have been performed to develop direct-acting antivirals (DAAs) for treating DENV infection^42^,^43^,^44^. However, the efficacy of DAAs is limited because of the high replication rate of DENV and low fidelity of NS5 RNA polymerase; this has resulted in the emergence of DAA-resistant variants^45^. In addition, different serotypes of DENV may affect the sensitivity of the virus to DAAs. In the present study, we clearly observed that HO-1 induction inhibited the replication of the 4 DENV serotypes (Fig. 1H). Therefore, targeting host factors exert harmful effects on the viral life cycle, such as HO-1, is a favorable strategy to prevent the emergence of DAA-resistant variants and genetic variability in the viral genome. In the present study, we have identified a potential natural product andrographolide with anti-DENV activity *in vitro* and *in vivo* (Figure S6 and Fig. 8), which can be considered as a promising drug or dietary supplement for the treatment of DENV-infected patients.

Several studies have shown that HO-1 exerts anti-inflammatory effects by inactivating nuclear factor-κB (NF-κB) and mitogen-activated protein kinases (MAPKs)^46^,^47^. NF-κB and MAPKs act as major inflammatory transcriptional factors that bind to consensus sequences on pro-inflammatory genes such as COX-2 and promote their expression^48^. Previous studies indicate that NF-κB- and MAPKs-mediated COX-2 activation may be used by some viruses such as HCV to promote viral replication^49^. In our previous study, we observed that potential agents inhibited HCV replication by inhibiting COX-2 expression or activation^50^,^51^. Therefore, we assumed that HO-1-NF-κB-COX-2 and HO-1-MAPKs-COX-2 pathways may serve as alternative mechanisms underlying the anti-DENV effect of HO-1 induction. The precise role of NF-κB- and MAPKs-mediated COX-2 activation in the anti-DENV effect of HO-1 should be elucidated in further studies.

Moreover, numerous studies have shown that ROS-induced oxidative stress is involved in the pathogenesis of a DENV infection and the progression of DHF and DSS^52^. ROS is eliminated by antioxidant molecules such as glutathione (GSH), superoxide dismutase, thioredoxin, and catalase, which comprise the cellular defense system that neutralizes oxidation and maintains a reductive intracellular environment^16^,^53^. GSH exerts cytoprotective effects against DENV-induced liver injury and alleviates the symptoms of DHF and DSS^16^.

The clinical course of dengue infection is divided into 3 distinct phases, in which the severe clinical manifestations (critical phase) are developed between day 3 to day 7^54^. In the present study, we observed that two specific HO-1 inducers extended the life-span of DENV-infected mice more than 6 days after a longer period of treatment (Figures S4 and S8), suggesting that HO-1 induction could become a potential tactic to aid DENV-infected patients passing through critical phase into recovery phase. Therefore, HO-1 may function as a potential antioxidant molecule and exert cytoprotective effects on DENV-associated cell damage and the life-threatening symptoms associated with DHF and DSS. However, further studies should be performed to investigate the effects of HO-1 induction on the pathogenesis of DENV infection. In summary, our results showed that HO-1 induction markedly inhibited the DENV replication through inhibiting DENV protease and restoring an antiviral IFN response, which can be used for future developing a potential therapeutic strategy and designing anti-DENV agents.

## Material and Method

### Ethics statement and experimental animals

Six-days-old ICR suckling mice were used in this study and breeder mice of the ICR strain were obtained from BioLasco Taiwan Co. Ltd. All animal studies were conducted in specific pathogen-free conditions and methods were carried out in accordance with the Guide for the Care and Use of Laboratory Animals. The experimental protocol were approved by the Animal Research Committee of Kaohsiung Medical University of Taiwan (IACUC, 102177) under the guidance of the Public Health Service (PHS) Policy on Humane Care and Use of Laboratory Animals. All mice received humane care and were fed with standard rodent chew and water ad libitum. Mice were acclimatized under a standard laboratory condition following the Animal Use Protocol of Kaohsiung Medical University for a week before experiment.

### Cells and virus

Huh-7 cells were maintained in Dulbecco’s modified Eagle’s medium (DMEM) containing 10% fetal bovine serum, 1% non-essential amino acids, and 1% antibiotic-antimycotic within 5% CO_2_ supplement at 37 °C. DENV (type 2 strain 16681) was amplified in C6/36 mosquito cells^55^. DENV of different serotypes (DENV-1: DN8700828; DENV-2: DN454009A; DENV-3: DN8700829A; DENV-4: S9201818) were obtained from the Centers for Disease Control, Department of Health, Taiwan.

### Reagents

CoPP, SnPP, hemin, Methylene chloride, Iron(III) chloride and andrographolide were obtained from Sigma (St. Louis, MO, USA). Biliverdin was obtained from MP Biomedicals (Heidelberg, Germany). T-Pro™ transfection reagent was obtained from (Ji-Feng Biotechnology Co., Ltd., Taipei, Taiwan).

### Plasmid construction

pCMV-HO-1-myc, an expression vector containing HO-1 gene fusion with myc taq under the control of cytomegalovirus (CMV) promoter. pISRE-Luc, a reporter vector containing firefly luciferase under the control of an IFN-stimulated response element (ISRE) (Stratagene, Agilent Technologies, CA). Biliverdin reductase (BVR) and enhanced green fluorescent protein (EGFP) shRNA were purchased from the National RNAi Core Facility, Institute of Molecular Biology/Genomic Research Center, Academia Sinica, Taiwan. The pNS2B(H)-NS3pro plasmid contains DENV serotype 2 NS2B hydrophilic domain (residue 43–95) and the NS3 protease domain (residue 1–187) which linked with native NS2B/NS3 polyprotein cleavage junction as described before^40^. The NS2B hydrophilic domain is defined as NS2B(H), and the NS3 protease domain is defined as NS3pro. In brief, The NS2B(H) and NS3pro were amplified by polymerase chain reaction (PCR) from cDNA of DENV and the NS2B(H) part and NS3pro part were linked by native NS2B/NS3 polyprotein cleavage junction and cloned into pcDNA4/Myc-His A (Invitrogen) vector. The pEG(∆4B/5)NLuc reporter vector contains enhanced green fluorescent protein (EG)-nano luciferase (NLuc) fusion protein which are linked by a DENV protease cleavage peptide sequences, TTSTRR-GTGNIG, based on NS4B/NS5 native polyprotein cleavage junction. The EG gene was amplified by PCR from pEGFP-C1 (Clontech), and the NLuc gene was amplified from pNL2.3[secNluc/Hygro] vector (Promega). The pNS2B(G_4_SG_4_)NS3 plasmids contains DENV NS2B(H) (residue 49–92) and NS3pro (residue 1–184) which were linked by a flexible glycine linker (Gly_4_-Ser-Gly_4_) for a non-autocleavage protease activity^56^. Briefly, the NS2B(H) and the NS3pro were amplified by PCR from cDNA of DENV. All cloned DNA fragments were verified by DNA sequencing.

### Western blotting

Western blotting was performed as described previously^57^. In brief, equal loading of cell lysates were analyzed by SDS-PAGE, followed by transfer to PVDF membrane. Membrane samples were probed with antibody specific for anti-DENV NS2B antibody (1:3,000; GeneTex), anti-GAPDH antibody (1:10,000; GeneTex), anti-biliverdin reductase (BVR) (1:3000, Abcam), anti-Myc antibody (1:2,000; Abcam), and anti-HO-1 antibody (1:3000; Abcam).

### DENV and cellular mRNAs RNA quantification

Total cell RNA was extracted by RNA extraction kit (GeneMark biolab Co, Ltd, Taiwan) following manufacturer’s instrument. DENV RNA and cellular mRNAs level were analyzed by quantitative real-time reverse-transcription polymerase chain reaction (RT-qPCR) as previously described^50^. Relative DENV RNA level was normalized against cellular glyceraldehydes-3-phosphate dehydrogenase (*gapdh*) mRNA. Primers used in the study are listed in Table S1.

### Immunofluorescence assay

The Huh-7 cells were plated in 24-well plates at 5 × 10^4^ per well and infected by DENV at 0.2 MOI for 2 hours and followed by CoPP treatment for 3 days. After treatment, the cells were washed by PBS twice. Subsequently, cells were fixed with 4% paraformaldehyde for 20 minutes and followed with 0.2% Triton X-100 for 20 minutes. The fixed cells were blocked in 4% BSA for 1 hour, followed by incubation of anti-prM antibody (1:500, GeneTex, Irvine, CA) for 1 hour. The cells were washed with PBS for 6 times and followed with Alexa Flour 488-conjugated goat anti-rabbit antibody (1:500, Life Technologies Corporation) for 1 hour. The cells were washed with PBS for 6 times and counterstained with DAPI (1:3000, Life Technologies Corporation) for 5 minutes. The cells were washed with PBS for 6 times and observed with microscope.

### Transfection and luciferase activity assay

To evaluate the transcriptional regulation of IFN response by HO-1 induction, Huh-7 cells were plated in 24-well plates at 5 × 10^4^ per well and transfected with pISRE-Luc plasmids using T-Pro™ transfection reagent according to the manufacturer’s instructions. The transfected cells were treated with indicated compounds for 3 days. Luciferase activity was analyzed by Steady-Glo Luciferase Assay System (Promega, Madison, WI) as previously described^51^. To evaluate the role of biliverdin reductase on DENV replication, biliverdin reductase shRNA expression vector pBVR-shRNA was transfected into the DENV-infected Huh-7 cells, followed by incubation with 50 μM biliverdin for 3 days. The cells were collected for western blotting and RT-qPCR assay.

### Expression and purification of NS2B-NS3pro

The pET24b-NS2B-NS3pro plasmid was transformed into competent Escherichia coli BL21 (DE3). The transformed competent cells were grew in 1 L LB medium containing kanamycin (50 μg/ml) at 26 °C until OD_600_ nm reached 0.6. The protein induction was performed through adding isopropyl-β-D-thiogalactopyranose to bacterial culture at 0.5 mM for 12 h. Cells were harvested and were suspended with binding buffer (20 mM sodium phosphate, pH 7.4, 500 mM NaCl, 10 mM imidozole). After sonication, the cleared lysates were subjected to metal chelation column chromatography using Ni-NTA His-bind resin. The column was washed with 15 column volumes of the lysates of wash buffer (20 mM sodium phosphate, pH 7.4, 500 mM NaCl, 40 mM imidazole). The bound NS2B-NS3pro proteins were eluted with 40 ml of elute buffer (20 mM sodium phosphate, pH 7.4, 500 mM NaCl, 250 mM imidazole). The fraction containing NS2B-NS3pro protein was collected and condensed by Amicon Ultra-15 10 K (Millipore).

### DENV NS2B/NS3 protease assays

Enzyme-based DENV NS2B/NS3 protease assay was performed as described previously^58^. In brief, analysis of DENV NS2B/NS3 protease activity was performed by using the 7-amino-4-methylcoumarin (AMC) fluorophore-linked peptide substrate Boc-GRR-AMC (Bachem, USA) and purified DENV NS2B-NS3pro protein. The indicated concentrations of biliverdin were incubated with 100 nM DENV NS2B-NS3pro and 10 μM Boc-GRR-AMC in cleavage buffer (200 mM Tris [PH 9.5], 20% glycerol) for 30 min at 25 °C. The fluorescence of released AMC, which was emitted at 465 nm following excitation at 380 nm, were detected by FL3-22 spectrofluorometer (Horiba Jobin Yvon). To evaluate the cis-acting activity of DENV NS2B/NS3 protease in Huh-7 cells, Huh-7 cells were plated in 24-well plates at 5 × 10^4^ per well and transfected with 1 μg of DENV NS2B/NS3 protease vector pNS2B(H)-NS3pro with autocleavage activity, the transfected-cells were treated with biliverdin at concentrations of 25, 50 and 100 μM for 3 days. The western blotting analysis were performed to examine the amount of uncleaved and cleaved form of NS2B/NS3 protein using anti-DENV NS2B specific antibody. To evaluate the trans-acting activity of DENV NS2B/NS3 protease in Huh-7 cells, Huh-7 cells were plated in 24-well plates at 5 × 10^4^ per well and co-transfected with 0.25 μg of NS2B/NS3 protease reporter vector pEG(∆4B/5)NLuc and 0.75 μg of non-autocleavage NS2B/NS3 protease vector pNS2B(G4SG4)NS3. The transfected-cells were incubated with biliverdin at concentrations of 25, 50 and 100 μM for 3 days. Supernatants were harvested for the nano luciferase activity assay using the Nano-Glo® Luciferase Assay System (promega) Each transfection mixture contained 0.1 μg of firefly luciferase expression vector (pCMV-FLuc) as a transfection control for normalization against the nano luciferase activity. Transfection of dysfunctional NS2B/NS3 protease expression vector, pNS2B/NS3^mut^, carrying double point mutations (L75A/S135A) in protease activity site served as a negative control. To evaluate the trans-acting activity of DENV NS2B/NS3 protease in DENV-infected Huh-7 cells, the DENV-infected Huh-7 cells were transfected with 1 μg of NS2B/NS3 protease reporter vector pEG(∆4B/5)NLuc. Then the cells were treated with biliverdin at increasing concentrations for 3 days. Supernatants were harvested for the nano luciferase activity assay using the Nano-Glo® Luciferase Assay System (promega). Each transfection mixture contained 0.1 μg of firefly luciferase expression vector (pCMV-FLuc) as a transfection efficacy control for normalization against the nano luciferase activity.

### Determination of the inhibition constant of biliverdin against DENV NS2B/NS3 protease

To determine the inhibition constant of biliverdin against DENV NS2B/NS3 protease, 100 nM DENV NS2B-NS3 protease was incubated with Boc-GRR-AMC concentrations ranging from 0 to 5 μM and increasing concentrations of biliverdin (5, 12.5 and 25 μM) in cleavage buffer for 30 min at 25 °C. The fluorescence readout was detected by FL3-22 spectrofluorometer. The Ki and Km values and the mechanistic mode of NS2B/NS3 inhibition was calculated from the Lineweaver–Burk plot and Michaelis–Menten equation^59^,^60^.

### Anti-DENV activity assay *in vivo*

Six-days-old ICR suckling mice were randomly divided into 3 groups (n = 10/group): Group 1: intracerebrally injected 2.5 × 10^5^ pfu 60 °C heat-inactive DENV (iDENV). Group 2: intracerebrally injected 2.5 × 10^5^ pfu DENV + intraperitoneally injected saline (DENV). Group 3; intracerebrally injected 2.5 × 10^5^ pfu DENV virus + intraperitoneally injected 50 mg/kg CoPP (DENV + CoPP 50 mg/kg) or 10 mg/kg andrographolide (DENV + Andrographolide 10 mg/kg). For 6 days assessment, the DENV-infected mice were given the tested agents by intraperitoneal injection at 1, 3, 5 days post-infection (dpi). For 12 days assessment, the DENV-infected mice were given the tested agents by intraperitoneal injection at 1, 3, 5, 7 and 9 dpi. The survival rate, body weight and clinical score were measured every day after DENV injection. Illness symptom were scored ad follow; 0 for no symptom; 1 for slight losing weight and ruffled hair; 2 for slowing of activity; 3 for asthenia; 4 for paralysis and mortally ill; 5 for death. At 6 dpi the mice were sacrificed by CO_2_ asphyxiation. 0.1 g brain tissue were harvested by R.I.P. A buffer, TRIzol and RPMI medium for protein, RNA and virus collection, respectively. The brain tissues were collected, weighed and homogenized in 500 ml of RPMI medium. The supernatant was collected by centrifugation at 8000 rpm for 15 min at 4 °C and frozen at −70 °C for plaque assay.

### Plaque assay

BHK-21 cells were plated in 12-well plates at 1 × 10^5^ per well. The virus collected from mice brain was serially diluted and was incubated with BHK-21 cells in a volume 400 μl at 30 °C. After 2 h incubation, the 3 ml DMEM containing 2% FBS and 0.8% methyl cellulose (Sigma-Aldrich) were added into each well. At 5 days post-infection, cells were fixed and stained with the plaque assay solution (1% crystal violet, 0.64% NaCl, and 2% formalin) at 25 °C for 2 h. Finally, the viral titer was calculated by observation of plaque formation^61^.

### Statistical analysis

The data were expressed as mean ± SD of at least three independent experiments. Statistical calculations were analysed by the Student’s *t*-test; *p*-values < 0.01 were considered statistically significant.

## Additional Information

**How to cite this article**: Tseng, C.-K. *et al.* Human heme oxygenase 1 is a potential host cell factor against dengue virus replication. *Sci. Rep.* **6**, 32176; doi: 10.1038/srep32176 (2016).

## Supplementary Material

Supplementary Information
