# Supplementary material for: Human heme oxygenase 1 is a potential host cell factor against dengue virus replication
Source: Sci Rep. 2016 Aug 24;6:32176. doi: 10.1038/srep32176 (PMC4995454; doi:10.1038/srep32176)
Supplement: Supplementary Information [file srep32176-s1.pdf]

Human heme oxygenase 1 is a potential host cell factor against dengue virus replication.

Chin-Kai Tseng[1,2], Chun-Kuang Lin[3], Yu-Hsuan Wu[1], Yen-Hsu Chen[4,5,6,7], Wei-Chun Chen[8], Kung-Chia Young[1,2,9*], Jin-Ching Lee[10,11,12*]

[1] Institute of Basic Medical Sciences, College of Medicine, National Cheng Kung University, Tainan, Taiwan

[2] Center of Infectious Disease and Signaling Research, College of Medicine, National Cheng Kung University, Tainan, Taiwan

[3] Doctoral Degree Program in Marine Biotechnology, College of Marine Sciences, National Sun Yat-Sen University, Kaohsiung, Taiwan

[4]Division of Infectious Diseases, Department of Internal Medicine, Kaohsiung Medical University Hospital, Kaohsiung, Taiwan

[5]School of Medicine, Graduate Institute of Medicine, Sepsis Research Center, Center for Dengue Fever Control and Research, Kaohsiung Medical University, Kaohsiung, Taiwan

[6]Department of Biological Science and Technology, College of Biological Science and Technology, National Chiao Tung University, HsinChu, Taiwan

[7]Center for Dengue Fever Control and Research, Kaohsiung Medical University, Kaohsiung, Taiwan.

[8]Graduate Institute of Medicine, College of Medicine, Kaohsiung Medical University, Kaohsiung, Taiwan.

[9]Department of Medical Laboratory Science and Biotechnology, College of Medicine, National Cheng Kung University, Tainan, Taiwan

[10]Department of Biotechnology, College of Life Science, Kaohsiung Medical University, Kaohsiung, Taiwan

[11]Graduate Institute of Natural Products, College of Pharmacy, Kaohsiung Medical University, Kaohsiung, Taiwan

[12]Research Center for Natural Products and Drug Development, Kaohsiung Medical University, Kaohsiung, Taiwan

Running title: Heme oxygenase 1 against dengue virus

***Corresponding authors:** Jin-Ching Lee and Kung-Chia Young

Mail address: Department of Biotechnology, Kaohsiung Medical University, 100, Shih-Chuan 1st Road, San Ming District, 807 Kaohsiung City, Taiwan.

Phone: 886-7-312-1101 ext. 2369 **Fax:** 886-7-312-5339

E-mail: jclee@kmu.edu.tw and t7908077@mail.ncku.edu.t

Supplementary material and method

Cell cytotoxicity assay

Huh-7 cells were plated in 96-well plates at 5×10^3 per well and treated with chemicals at indicated conditions. The cell viability was determined by the CellTiter 96 AQ$_{ueous}$ One Solution Cell Proliferation Assay (Promega, WI, USA) according to the manufacturer's instructions. Color intensity was detected at 490 nm using a 550 BioRad plate-reader (Bio-Rad, Hertfordshire, UK).

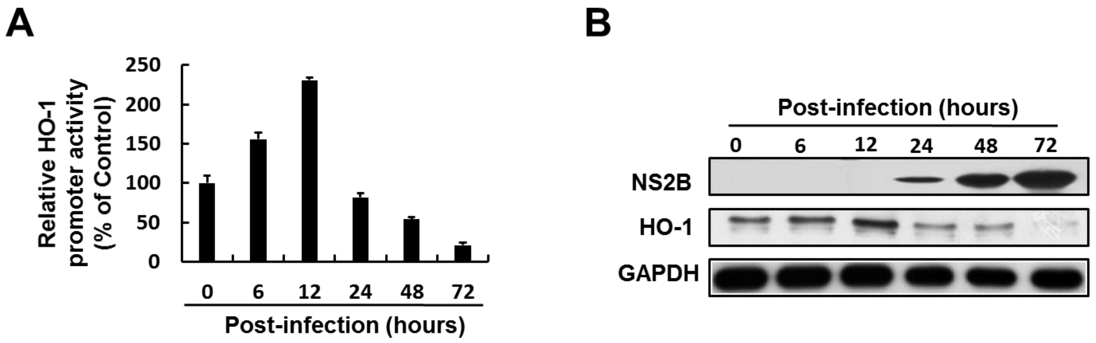

Figure S1. Time course analysis of HO-1 (A) promoter activity and (B) protein expression influenced by DENV infection.

Huh-7 cells were co-transfected with 0.5 μg of pHO-1-Luc and 0.1 μg of pCMV-renilla, and followed by DENV infection at an M.O.I. of 5. The values in promoter activity are expressed as a relative percentage change compared with zero time-point of infection (defined as 100%) following normalization of Renilla luciferqase activity. The HO-1 protein expression was measured by western blotting with anti-DENV NS2B, HO-1 and GAPDH antibodies. Error bars denote the means ± SD of 3 independent experiments.

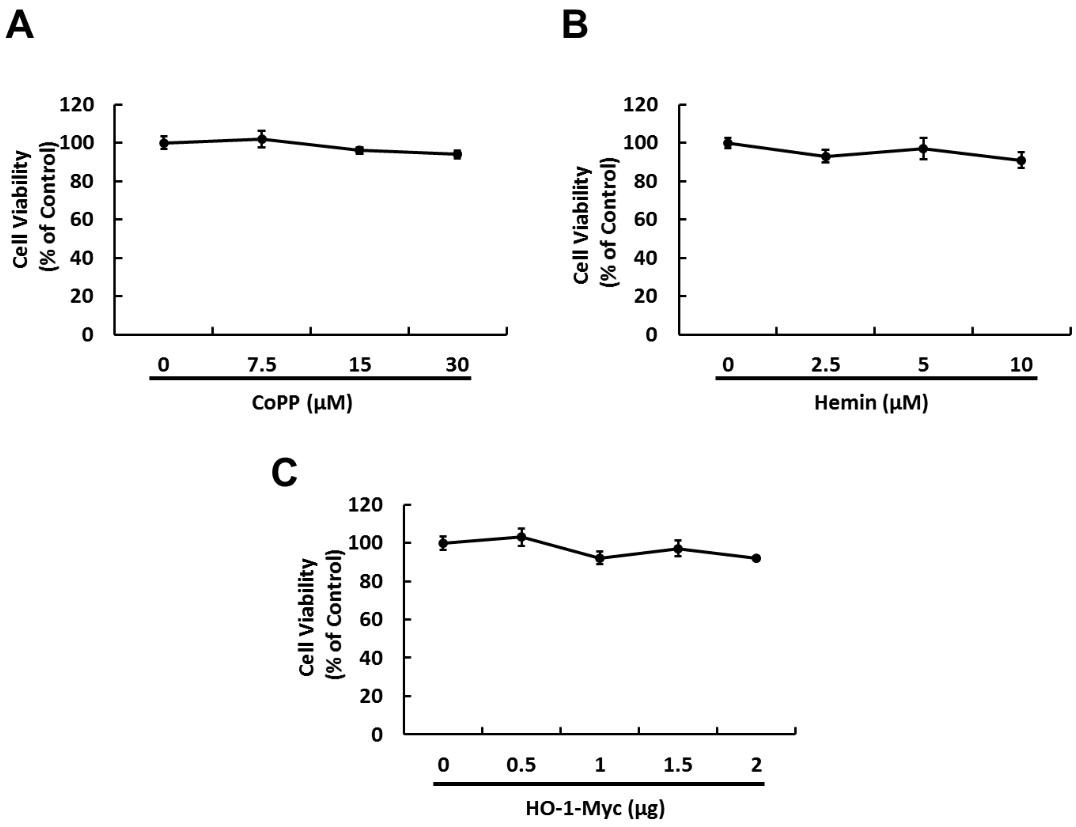

Figure S2. Effect of CoPP and hemin on the cell viability of Huh-7 cells.

Huh-7 cells were plated 5×10^3 per well in the 96-well plate and treated with CoPP or hemin at the indicated concentrations for 3 days. Cell viability was measured by the CellTiter 96 AQ_{ueous} One Solution Cell Proliferation Assay. Error bars indicate the means ± SD of 3 independent experiments. **P* < 0.05; ***P* < 0.01.

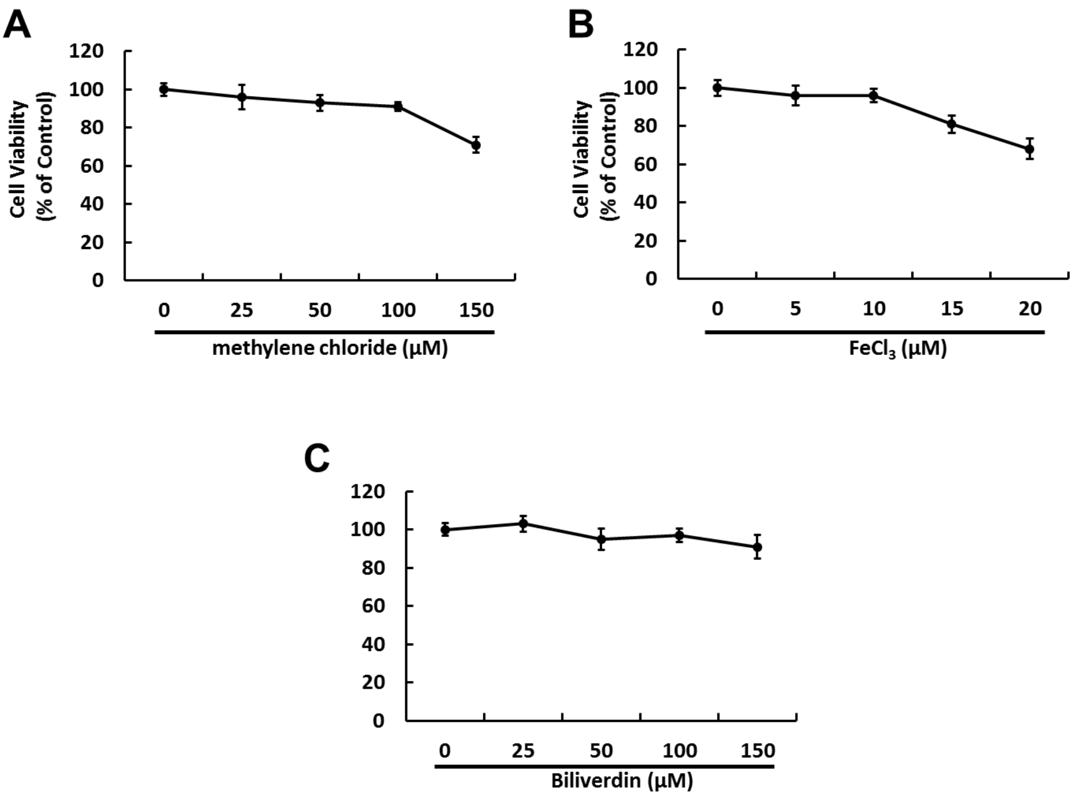

Figure S3. Effect of Methylene chloride, Iron(III) chloride and biliverdin on the cell viability of Huh-7 cells.

Huh-7 cells were plated 5×10^3 per well in the 96-well plates and treated with Methylene chloride, Iron(III) chloride or biliverdin at the indicated concentrations for 3 days. Cell viability was measured by the CellTiter 96 AQ$_{ueous}$ One Solution Cell Proliferation Assay. Error bars indicate the means ± SD of 3 independent experiments. *$P < 0.05$; **$P < 0.01$.

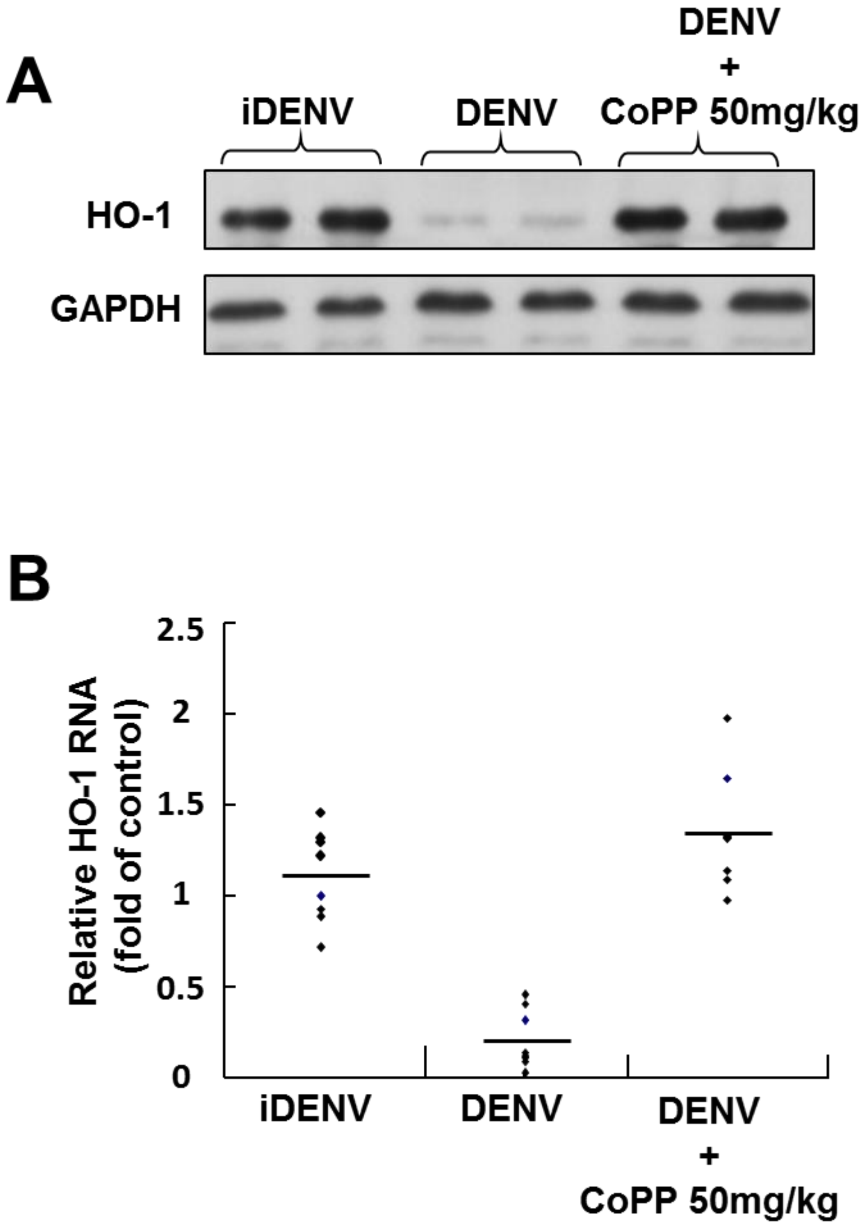

Figure S4. HO-1-specific inducer CoPP increases HO-1 expression in DENV-infected ICR suckling mice.

Six-day-old ICR suckling mice were injected with 2.5 × 10^5 pfu DENV-2 intracerebrally and with CoPP (50 mg/kg) intraperitoneally at 1, 3, and 5 dpi. Next, 0.1 g of brain tissue of these mice was lysed in RIPA lysis buffer, and treated with TRIzol reagent for protein extraction, and RNA extraction, respectively. (A) HO-1 protein

levels were measured by performing western blotting by using HO-1-specific antibody, with GAPDH as the equal loading control. (B) HO-1 mRNA level was measured by performing RT-qPCR with specific primers against the gene encoding HO-1. The mRNA level of HO-1 was normalized against that of cellular *gapdh* and was presented as fold change relative to that in non-CoPP-treated mice (defined as 1). Mice in the control group were treated with 60°C heat-inactive DENV for 30 min. Each group had 8–10 mice.

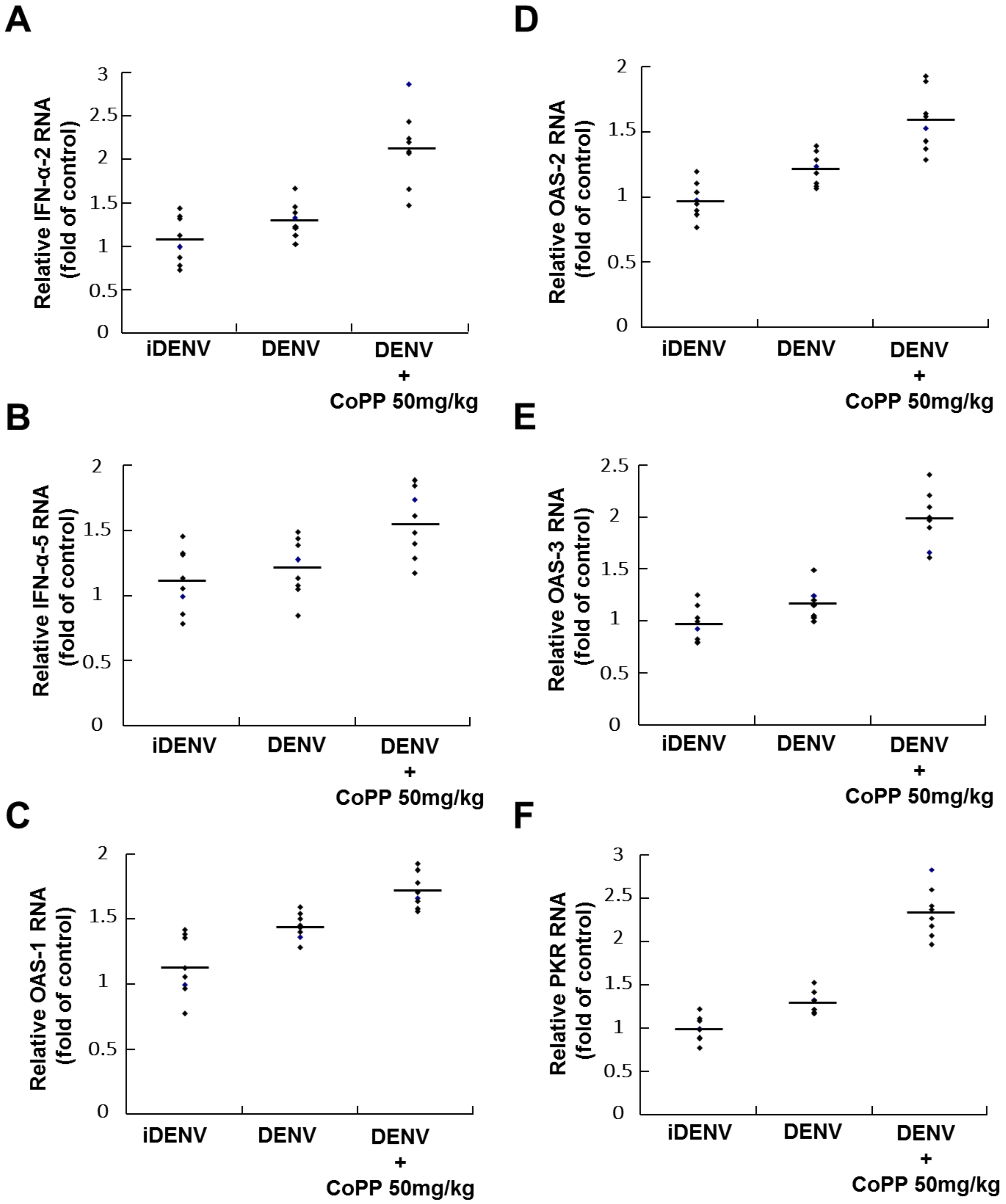

Figure S5. HO-1-specific inducer CoPP induces antiviral IFN responses in DENV-infected ICR suckling mice.

Six-day-old ICR suckling mice were injected with 2.5×10^5 pfu DENV-2 intracerebrally and with CoPP (50 mg/kg) intraperitoneally at 1, 3 and 5 dpi. Next, 0.1 g of brain tissue of these mice was treated with TRIzol reagent for RNA extraction. The

mRNA level of genes encoding (A) IFN-α-2, (B) IFN-α-5, (C) OAS1, (D) OAS2, (E) OAS3, and (F) PKR were measured by performing RT-qPCR with specific primers, were normalized against that of cellular *gapdh*, and were presented as fold change relative to that in non-CoPP-treated mice (defined as 1). Mice in the control group were treated with 60°C heat-inactive DENV for 30 min. Each group included 8–10 mice.

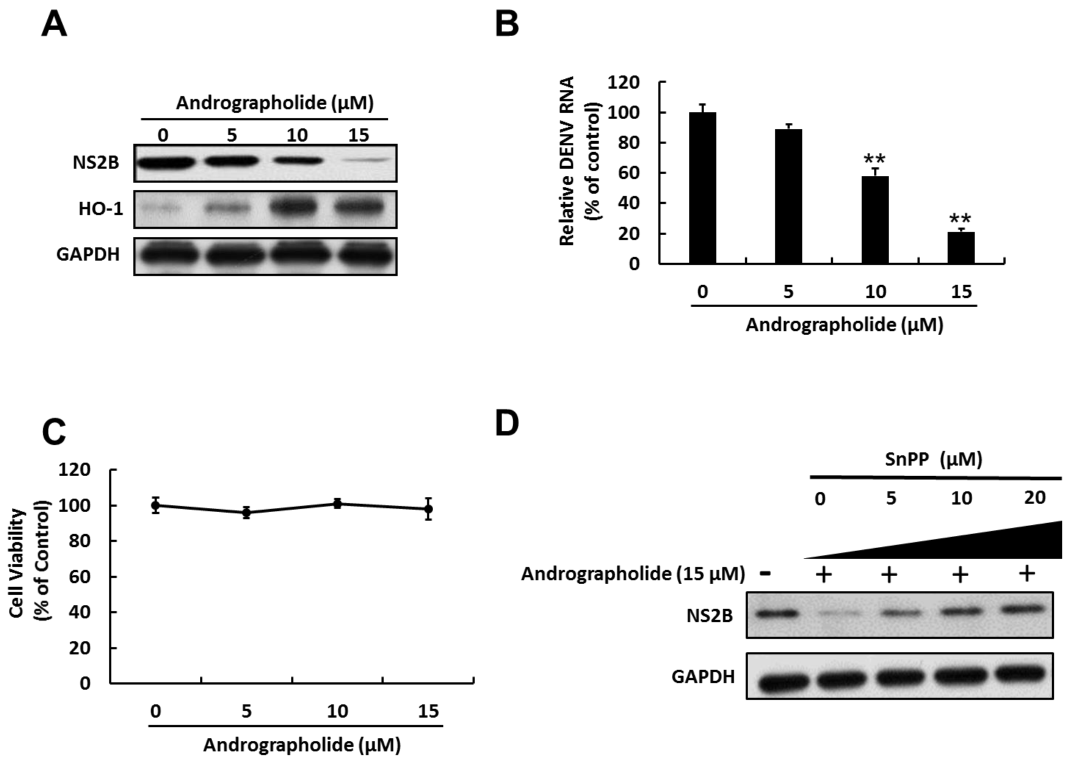

Figure S6. Andrographolide inhibits DENV replication via inducing HO-1 protein expression.

Andrographolide enhances (A) HO-1 protein synthesis and inhibits DENV protein synthesis and (B) RNA replication. DENV-infected Huh-7 cells were treated with andrographolide at 5, 10, 15 and 20 μM for 3 days. Western blotting assay was employed to examine the HO-1 and DENV protein expression. Levels of GAPDH were used as equal loading control. The DENV RNA level was normalized against that of cellular *gapdh* and were presented as percentage change relative to that in parental Huh-7 cells (defined as 100%). (C) Effect of andrographolide on the cell viability of Huh-7 cells. Huh-7 cells were plated 5×10^3 per well in the 96-well plates and treated with andrographolide at the indicated concentrations for 3 days. Cell viability was measured

by the CellTiter 96 AQ_{ueous} One Solution Cell Proliferation Assay. (D) SnPP rescues the inhibitory effect of andrographolide on DENV protein synthesis. DENV-infected Huh-7 cells were coincubated with 20 μM andrographolide and increasing concentrations of HO-1-specific inhibitor SnPP (0-20 μM) for 3 days. The total cells lysates were examined by performing western blotting to analyze DENV protein level. Levels of GAPDH were used as equal loading control.

Table S1. Oligonucleotide sequences for real-time RT-PCR

Oligonucleotide Name	Sequence 5'-3'
**DENV gene oligonucleotide sequences**	
5' NS5	5'-GGA AACCAAGCTGCCCATCA
3' NS5	5'-CCTCCACGGATAGAAGTTTA
**Human gene oligonucleotide sequences**	
5' GAPDH	5'-GTCTTCACCACCATGGAGAA
3' GAPDH	5'-ATGGCATGGACTGTGGTCAT
5' OAS1	5’- CAAGCTTAAGAGCCTCATCC
3' OAS1	5’- TGGGCTGTGTTGAAATGTGT
5' OAS2	5’- ACAGCTGAAAGCCTTTTGGA
3' OAS2	5’- GCATTAAAGGCAGGA AGCAC
5' OAS3	5’- CACTGACATCCCAGACGATG
3' OAS3	5’- GATCAGGCTCTTCAGCTTGG
5' PKR	5’- ATGATGGAAAGCGAACAAGG
3' PKR	5’- GAGATGATGCCATCCCGTAG
5' IFN-alpha 2	5'-GCA AGT CAA GCT GCT CTG TG
3' IFN-alpha 2	5’-GAT GGT TTC AGC CTT TTG GA

5' IFN-alpha 5	5’- AGTTTGATGGCAACCAGTTC
3' IFN-alpha 5	5’- TCAGAGGAGTGTCTTCCACT
5' IFN-alpha 17	5'-AGG AGT TTG ATG GCA ACC AG
3' IFN-alpha 17	5'-CAT CAG GGG AGT CTC TTC CA

Mice gene oligonucleotide sequences

5' GAPDH	5'-GTCTTCACCACCATGGAGAA
3' GAPDH	5'-ATGGCATGGACTGTGGTCAT
5' OAS1	5’- CAAGCTTAAGAGCCTCATCC
3' OAS1	5’- TGGGCTGTGTTGAAATGTGT
5' OAS2	5’- ACAGCTGAAAGCCTTTTGGA
3' OAS2	5’- GCATTAAAGGCAGGA AGCAC
5' OAS3	5’- CACTGACATCCCAGACGATG
3' OAS3	5’- GATCAGGCTCTTCAGCTTGG
5' PKR	5’- ATGATGGAAAGCGAACAAGG
3' PKR	5’- GAGATGATGCCATCCCGTAG
5' IFN-alpha 2	5'-GCA AGT CAA GCT GCT CTG TG
3' IFN-alpha 2	5’-GAT GGT TTC AGC CTT TTG GA
5' IFN-alpha 5	5’- AGTTTGATGGCAACCAGTTC
3' IFN-alpha 5	5’- TCAGAGGAGTGTCTTCCACT

5' IFN-alpha 17	5'-AGG AGT TTG ATG GCA ACC AG
3' IFN-alpha 17	5'-CAT CAG GGG AGT CTC TTC CA
